# Interactions between the transcription and replication machineries regulate the RNA and DNA synthesis in the herpesviruses

**DOI:** 10.1007/s11262-019-01643-5

**Published:** 2019-02-14

**Authors:** Zsolt Boldogkői, Dóra Tombácz, Zsolt Balázs

**Affiliations:** Department of Medical Biology, Faculty of Medicine, University of Szeged, Somogyi B. u. 4., Szeged, 6720 Hungary

**Keywords:** Herpesvirus, Transcriptional overlap, DNA replication, Transcriptional interference, Nanopore sequencing

## Abstract

The temporal coordination of viral gene expression is imperative for the regulation of the herpesvirus replication cycle. While the main factors of this transcriptional coordination are known, the subtler control mechanisms of gene expression remain elusive. Recent long read sequencing-based approached have revealed an intricate meshwork of overlaps between the herpesvirus transcripts and the overlap of the replication origins with noncoding RNAs. It has been shown that the transcriptional apparatuses can physically interfere with one another while transcribing overlapping regions. We hypothesize that transcriptional interference regulates the global gene expression across the herpesvirus genome. Additionally, an overall decrease in transcriptional activity in individual viral genes has been observed following the onset of DNA replication. An overlap of the replication origins with specific transcripts has also been described in several herpesviruses. The genome-wide interactions between the transcriptional apparatuses and between the replication and transcriptional machineries suggest the existence of novel layers of genetic regulation.

## Background

The herpesviruses are a large group of viruses that infect a wide-range of vertebrate organisms [1], and they are responsible for several human and veterinary diseases. Following the circularization of the herpes genome upon entering the nucleus, DNA synthesis is thought to proceed in two consecutive stages: an initial phase of θ-type replication is followed by σ-type replication. During the θ-type mechanism, the replication fork proceeds in two directions, whereas in the σ-type replication the progression of DNA replication machinery is unidirectional and includes a rolling-circle mechanism that generates concatemers. These multigenomic molecules are cleaved into unit genomes, which is followed by being packaged into the preformed empty capsids [2]. The viral life cycle is primarily regulated by the control of transcription. The viral genes are classified into three different kinetic groups: immediate-early, early, and late genes, which are defined by their peak rates of mRNA synthesis, and by how they behave in the presence of protein or DNA synthesis inhibitors. In all herpesvirus subfamilies, the transcription is regulated by a multitude of *cis-* and *trans-*acting elements [3, 4]. However, there are differences between the expression patterns of genes that belong to the same kinetic group [5–7], which are not explained by the function of known *cis-* and *trans-*acting elements. Modern molecular biology offers several tools for the examination of transcriptional interactions. Not only can one examine specific transcripts using Northern blot or their expression rates using RT-qPCR, but RNA sequencing allows for the investigation of the whole transcriptome without prior knowledge about the sequence. Auxiliary methods such as global run-on sequencing can give a more direct insight into the regulation of transcription [8, 9]. Short-read sequencing (SRS) technologies have limited capability in identifying multi-spliced transcripts, to distinguish between overlapping transcripts, and to detect multigenic transcripts [10]. The emerging long-read sequencing (LRS) can overcome these problems through its greater efficiency in identification of transcript isoforms, as well as polycistronic and overlapping transcripts.

## Genome-wide transcriptional overlaps

**Fig. 1:**
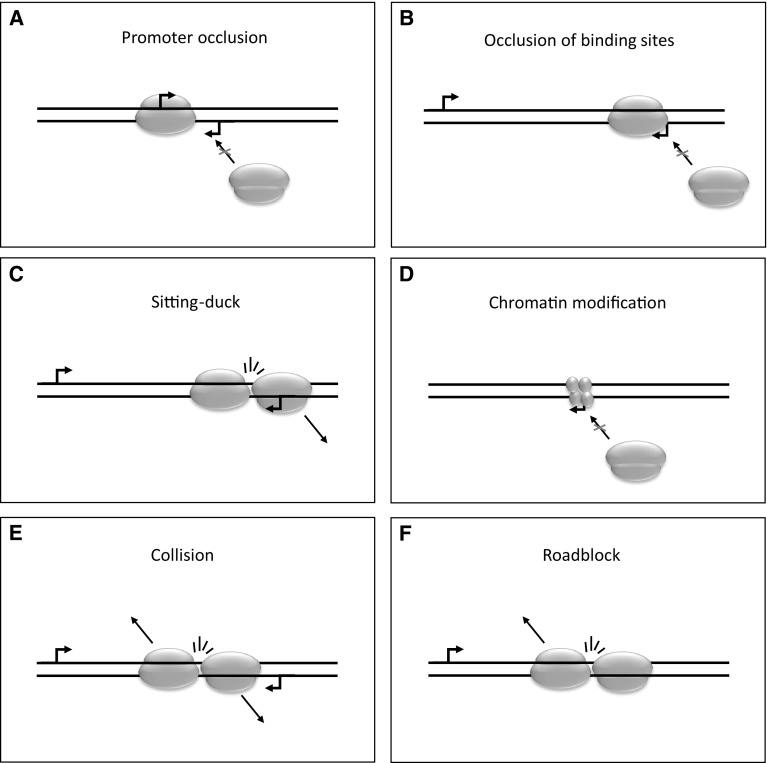
Models of transcriptional interference. Promoter occlusion may occur if two promoters are in the nigh vicinity of each other and the assembly of a transcriptional apparatus at one promoter blocks the assembly at the other one (**a**). Assembly can also be suppressed if progressing RNAPs block transcription factor binding sites (**b**). The sitting duck phenomenon describes the dislocation of an assembling transcription apparatus by a progressing RNAP (**c**). Chromatin modifications can also inhibit transcriptional initiation (**d**). Collision of the transcriptional apparatuses can occur in overlapping transcripts and is thought to result in the dislocation of both RNAPs (**e**). If an RNA polymerase is tightly bound to a genomic sequence, it can also create a roadblock, preventing any transcription from passing through (**f**)

The recent LRS-based investigations of herpesvirus transcriptomes have revealed an intricate meshwork of transcriptional overlaps in each subfamily of herpesvirus. In some cases, the transcriptional apparatus fails to recognize the polyadenylation signal, and as a result, the transcription continues beyond the original transcription termination site. It is a general phenomenon in cellular organisms that the RNA polymerase (RNAP) continues transcription for some distance beyond the poly(A) site before it is released from the DNA [11]. This may cause otherwise non-overlapping convergent genes to overlap (tail-to-tail overlap). Divergent herpesvirus genes in most cases overlap each other (head-to head overlap) at their promoters or more frequently at their transcribed regions. Most of the herpesvirus genes are organized into polycistronic transcription units, the members of which share common poly(A) signals (tail-to-head overlap). Long-read RNA sequencing techniques were able to identify long complex transcripts with genes in opposite orientations [12–14]. We propose that the transcriptional overlaps came into existence in order to create a genetic regulatory mechanism that controls gene expression through the interference between the transcriptional apparatuses of adjacent and distal genes. Transcriptional extension of the convergent genes or the overlapping of divergent genes can affect both the initiation and the elongation of transcription of two or more partners through transcriptional interference (TI) [15, 16]. Transcription initiation can be blocked by promoter competition when occupation of one promoter by RNAP precludes another RNAP from binding to occupation of the other promoter, thereby inhibiting the assembly of the transcription initiation complex for the transcription of the other gene. As the genes in herpesviruses are tightly packed, promoters are inevitably found adjacent to each other. For instance, the promoters of the divergent HSV-1 genes *ul37* (early-late) [17] and *ul38* (true late) [18] genes are found in a distance of less than 200 base pairs of each other and LRS has revealed a longer transcript isoform of *ul37* which initiates merely 71 nucleotides downstream of the *ul38* start site [12]. Due to their close proximity, the transcriptional apparatus as it is being assembled on one promoter or during transcription might prevent RNAP from binding to the other promoter (Fig. 1a, b). The initiation of transcription can also be obstructed by occlusion of binding sites by the progressing RNAPs [19], or through the ‘sitting-duck’ interference, when an elongating RNAP removes the other one that is already bound to its own promoter [20] (Fig. 1c). Histone coverage may impede transcription [21] (Fig. 1d); however, if continued transcription of a nearby locus keeps the DNA devoid of histones, it may induce transcription from a dormant promoter. Microarray hybridization has detected large transcripts spanning several genes in, e.g. KSHV [22], which means that not only directly neighbouring genes, but also genes separated by a dozen other genes, can be connected by transcripts. The transcription elongation can be inhibited by the collision of the progressing RNAPs resulting in a premature termination of transcription of one or both genes [23] (Fig. 1e), or by the ‘roadblock’ (polymerase pausing) mechanism, when one RNAP molecule becomes immobile, and therefore inhibits the progression of the transcription elongation complex coming from the other gene [24] (Fig. 1f). Such convergent transcription allows cell lines J-Lat 9.2 and 15.4 to silence the expression of the HIV provirus and keep it in latency [25]. It has been postulated that the antisense latency transcripts of herpesviruses [26, 27] may also silence the sense transactivator genes by transcriptional interference. Altogether, in each of the above cases, the transcriptions of the interacting partners exert a negative effect on each other’s activity; however, positive regulation is also possible through the inhibition of the inhibitory effects of other interactors. Transcription interference has only rarely been investigated in vivo; however, its powerful effects have been clearly demonstrated on synthetic constructs [28]. Examining the effects of transcriptional interference on a whole transcriptome level poses further challenges due to the large number of interactions that are to be considered. The combination of all TI events in a genome may be an emergent property, a transcriptional interference network (TIN) that is capable of fine-tuning the expression of each gene in a manner that would otherwise only be feasible using a handful of *cis*- and *trans*-acting elements.

## Interactions between the transcription and replication machineries

**Fig. 2:**
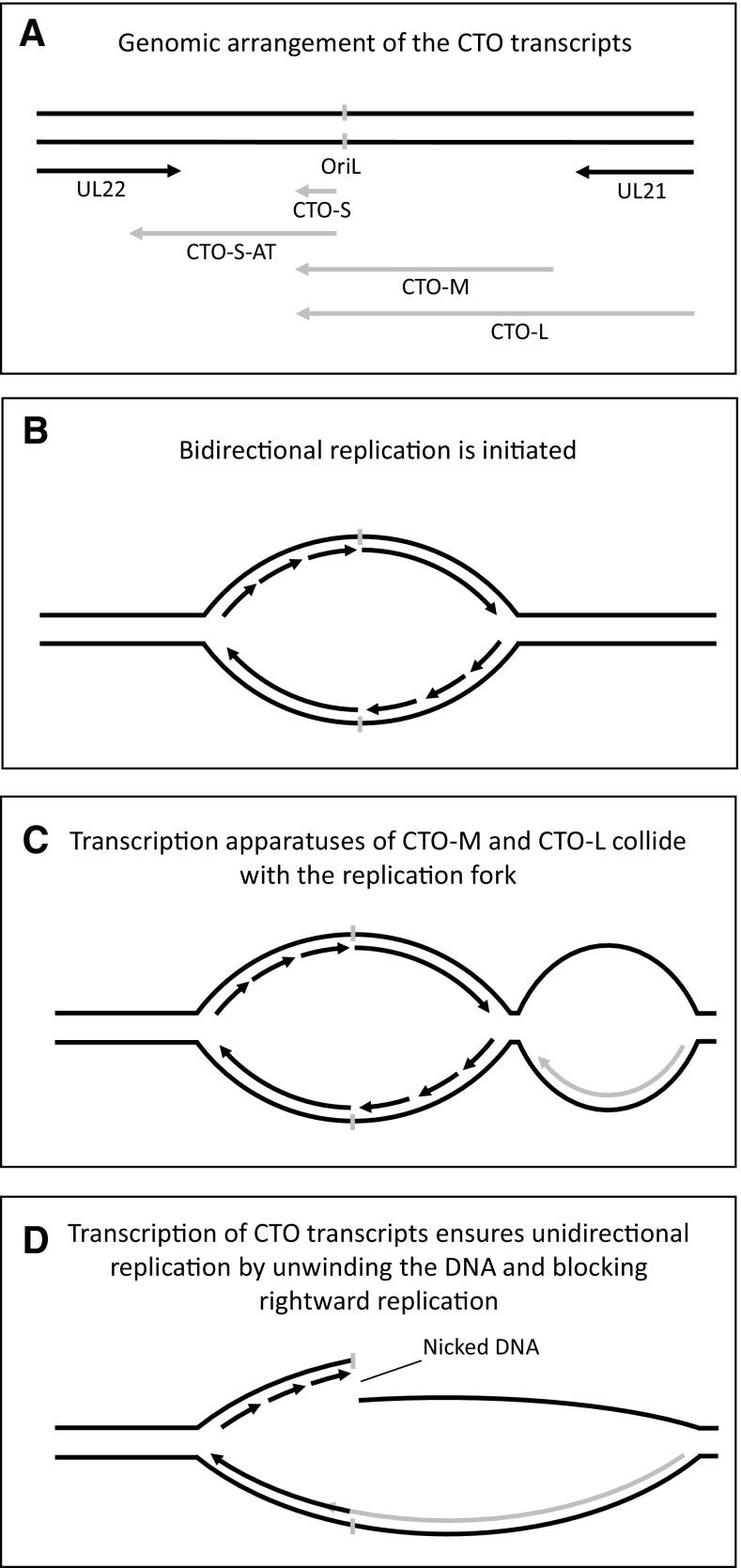
Putative regulatory role of CTO transcripts in PRV replication. **a** A schematic representation of the genomic segment surrounding the OriL (marked by a grey bar) of PRV. Transcripts are depicted by arrows. At first the genome is replicated through theta replication (**b**), however as the CTO transcripts become transcriptionally active, the replication fork and the transcriptional apparatus collide (**c**). The continued expression from the CTO transcripts represses DNA replication in one direction and facilitates it in the other, by opening the DNA strands (**d**)

Herpesvirus replication produces long concatemeric DNA. Replication has been thought to proceed in a rolling circle mechanism, similarly to the replication of λ phages; however, some evidence suggests that the replication of herpesviruses may be more complex and that concatemers may be formed by recombination [29]. Two observations give rise to the possibility of the interaction between the processes of translation and transcription in herpesviruses. The first is that the general expression rate of the transcription of individual viral genomes drops following the onset of replication [30]. The different kinetics of IE, E and L genes are explained by the differential effect of the transcription factors on the expression of these genes. For example, the herpesvirus immediate-early protein can downregulate the expression of their own expression [31, 32]. It cannot be ruled out that early proteins exert a similar negative feedback or that switch to late transcription results in the reduction of transcription during the initiation of viral replication. However, it is also possible that the replication itself also contributes to the control of gene expression kinetics. Another observation is the production of noncoding RNAs overlapping the replication origins (Oris) in many herpesviruses [12, 33–36]. The CTO-S is the most abundant pseudorabies virus (PRV) transcript and is first expressed at 4 h postinfection (when DNA replication commences); the other two co-terminal CTO transcripts are low-abundance RNAs. We hypothesize that the transcription of CTO-M and CTO-L collides with the DNA replication (at the OriL) progressing in a θ-type manner, and as a result, it renders the replication to being unidirectional (σ-type). The same may be the function of the PRV PTO-US1 (colliding with the replication fork at the OriS) [34]. In the meantime, CTO-S expression may separate the DNA strands, thereby further helping the unidirectional progression of the replication. The expression of CTO-AT is supposed to reduce the expression of the convergent *ul22* gene, thereby further helping the advance of DNA synthesis in a σ-type manner. It has been proposed that the CTO transcripts play a role in the switch from the θ-type to the σ-type of replication (Fig. 2) [33, 36]. Alternatively, this CTO-based mechanism blocks the initiation of bidirectional replication. In this scenario, no θ-type replication occurs. It is also possible that the Ori-overlapping transcripts direct the replication by forming R-loops (as reviewed by Lombraña et al. [37]). In the herpes simplex virus (HSV), two 5′-coterminal transcripts are expressed in the OriS region. Ori_s_RNA_1_ is expressed with early kinetics, while Ori_s_RNA_2_ is a late transcript [33]. Contrary to alphaherpesviruses, the human cytomegalovirus (HCMV) OriLyt region does not express abundant polyadenylated transcripts [13, 38]; however, a short non-polyadenylated RNA [35] is expressed from 2 h p.i. The Kaposi’s sarcoma-associated herpesvirus (KSHV) T1.5 (K4.7 or OriLyt transcript) is an early [39], polyadenylated transcript, which is indispensable for DNA replication [40]. A similar dependence of Epstein–Barr virus (EBV) replication on Zta-induced transcription has also been described [41]. The differences between the sizes and expression patterns of the different Ori-overlapping transcripts and their posttranscriptional modifications suggest that they might influence viral replication through different mechanisms. The phenomenon of the replication/transcription collision has been described in other systems [42]. The interplay between the DNA and RNA synthesis apparatuses is assumed to form a transcription and replication interference network (TRIN) that governs the global gene expression and the replication in a mutually interdependent manner.

## Conclusions

Recent transcriptomic studies have revealed a much greater functional diversity of the viral genome than it had been thought before. These studies have uncovered an extensive overlapping pattern of transcriptions in herpesviruses. The question can be raised as to whether the highly compact nature of viral genomes favours the evolution of this phenomenon, or if the function of transcriptional overlaps is to regulate gene expression through giving rise to transcriptional interference (TI). TI mainly act through the collision of or competition between the transcriptional apparatuses of adjacent or distal genes. The existence of polycistronic and complex transcripts suggests that transcriptional read-throughs are highly likely to have a function other than the translation of the downstream genes. The diversity of overlaps between the viral genes suggests that the TIs are organized into a system forming network, which may coordinate the viral life cycle in a spatiotemporal manner through the physical interaction of the transcriptional apparatuses. TI might have co-evolved with the factor-dependent regulation of gene expression [43, 44]. We consider TI as a system level property since practically each viral transcript overlap with other transcripts; therefore, changing the transcription of a gene can affect the expression of genes even at distal positions of the genome. It is hypothesized that these interactions may result in a well-controlled progression of the ON/OFF states of genes throughout the entire genome thereby generating a network of interactions, termed transcription interference network (TIN). The TIN is supposed to co-regulate the expression of genes through synchronization of the transcriptions. TIN forms a self-regulatory network, whose operation leads to a definite temporal pattern of genome-wide gene expressions. TIN is supposed to act to suppress the transcriptional noise, produced by the expression from genes whose gene products are unneeded at a given stage of the viral life cycle. DNA replication and transcription are also supposed to interact with one another at both the initiation and elongation phases of DNA replication. The transcription and replication interference network (TRIN) is also supposed to act on a system level because the onset of replication results to a global drop of gene expression in each gene on an individual DNA molecule, and because the progress of the replication fork is confronted with the transcription machineries along the entire viral genome.

## Abbreviations

SRS: Short-read sequencing
LRS: Long-read sequencing
TI: Transcriptional interference
RNA: PRNA polymerase
TIN: Transcriptional interference network
Ori: Replication origin
PRV: Pseudorabies virus
HSV: Herpes simplex virus
HCMV: Human cytomegalovirus
KSHV: Kaposi’s sarcoma-associated herpesvirus
EBV: Epstein–Barr virus
TRIN: Transcription and replication interference network
